# Identifying determinants and predicting cesarean section delivery among Bangladeshi women using machine learning: Insight from BDHS 2022 Data

**DOI:** 10.1371/journal.pgph.0005494

**Published:** 2025-11-19

**Authors:** Shamsuz Zoha, Shahin Alam, Isteaq Kabir Sifat, Nourin Sultana, Md. Kaderi Kibria

**Affiliations:** Department of Statistics, Hajee Mohammad Danesh Science and Technology University, Dinajpur, Bangladesh; McMaster University, CANADA

## Abstract

Cesarean section (C-section) rates have been rising globally, posing potential health risks for mothers and infants. Understanding the factors that contribute to C-section delivery and leveraging machine learning (ML) techniques for predictive modeling can support targeted interventions and informed policy decisions. This study aimed to identify the determinants of C-section delivery and develop an ML-based predictive model using data from the Bangladesh Demographic and Health Survey (BDHS) 2022. A total of 2,490 complete records of ever-married women aged 15–49 years were analyzed, where the delivery mode was categorized as vaginal or C-section. Three feature selection techniques including Recursive Feature Elimination (RFE), Boruta-based selection (BFS), and Random Forest (RF) were used to identify key risk factors. Six ML algorithms, including Logistic Regression (LR), Support Vector Machine (SVM), K-Nearest Neighbors (KNN), Decision Tree (DT), Random Forest (RF), and Extreme Gradient Boosting (XGB) were employed to predict C-section. Model performance was evaluated using accuracy, precision, recall, F1-score, AUC, and ROC analysis. SHAP values were used to interpret the influence of individual features.The prevalence of C-section deliveries was 45.6%, with an average maternal age of 25.7 years and mean age at first childbirth of 19.3 years. Ten significant determinants were identified, including place of delivery, baby weight, maternal BMI, birth interval, age at first birth, partner’s education, maternal age, wealth status, ANC visits, and maternal education. The RF model achieved the highest performance with an accuracy of 81.79%, and an AUC of 0.871. SHAP analysis highlighted that place of delivery, baby weight, maternal BMI, and birth interval were the most influential predictors. These findings suggest that socio-demographic and healthcare-related factors strongly influence C-section delivery. Machine learning models particularly the RF can effectively identify women at high risk, supporting strategies to reduce unnecessary C-sections and improve maternal healthcare planning in Bangladesh.

## Introduction

C-section delivery is a surgical procedure performed to deliver a baby through incisions made in the mother’s abdomen and uterus when vaginal delivery poses life-threatening risks to the mother or the baby due to medical complications [1,2]. While C-sections can be life-saving and have contributed to a decline in maternal mortality rates worldwide, unnecessary C-sections without valid medical justification can lead to severe adverse outcomes, including increased maternal and neonatal morbidity and mortality [3,4]. The World Health Organization (WHO) reports that more than one in five births are now delivered surgically, with projected increases in C-section rates globally by 2030, particularly in regions like Eastern Asia (63%), Latin America and the Caribbean (54%), and South Asia [5]. In Bangladesh, the prevalence of C-section delivery has surged drastically from 2.7% in 2000 to 45% in 2022, raising significant concerns about its overuse [6]. This rapid increase poses challenges such as post-delivery health risks, financial burdens on families, and, in some instances, severe complications for newborns [2].

Understanding the factors associated with C-section delivery is crucial to minimizing unnecessary procedures while ensuring access for those who need it. Socio-demographic factors, maternal characteristics, healthcare access behavior (such as antenatal care and place of delivery), and the mother’s personal preference often influence the decision for a C-section [7–10]. Previous studies have identified factors like maternal age, obesity, education, occupation, household income, and wealth status as significant predictors of C-section deliveries [11–17]. Additionally, disparities in C-section rates between private and public healthcare facilities have been documented, suggesting that institutional and healthcare access factors also play a role. Machine learning (ML) has gained increasing attention in medicine, with applications in predicting pre-eclampsia [18], intrauterine growth restriction [19], mother–newborn skin-to-skin contact [20], non-reassuring fetal heart patterns [21], and episiotomy risk [22]. These studies highlight the growing popularity and reliability of ML in healthcare, supporting its potential for maternal health research.

Although numerous studies have explored the prevalence and determinants of C-sections using conventional statistical methods, limited research has utilized advanced machine learning (ML) techniques to accurately predict the likelihood of C-section deliveries in Bangladesh. For instance, a recent study applied ML models including LR, RF, SVM, k-nearest neighbor (KNN), and naive Bayes (NB) to BDHS 2017–18 data, achieving an accuracy of 83.74% with NB [23]. However, these studies often relied on older datasets, lacked comprehensive feature selection techniques, and did not fully explore interpretability methods like SHAP analysis to understand the contributions of each predictor.

To address these gaps, this study aims to identify socio-demographic determinants of C-section deliveries and to develop robust machine learning models to predict the likelihood of C-section among Bangladeshi women using the nationally representative BDHS 2022 dataset. Additionally, this study aims to evaluate model performance. It also interprets key predictors using SHAP analysis and provides insights to inform clinical practice and policy decisions regarding C-section deliveries. Unlike previous studies, this research applies three advanced feature selection techniques RFE, BFS, and RF to ensure the most relevant predictors are considered. Furthermore, by employing multiple performance metrics such as precision, recall, F1-score, and SHAP analysis, this study seeks to provide more nuanced and actionable insights that can guide healthcare providers and policymakers in minimizing unnecessary C-sections and improving maternal health outcomes in Bangladesh.

## Materials and methods

### Ethics statement

This study analyzed publicly available data from the Bangladesh Demographic and Health Survey (BDHS) 2022 which is openly accessible online with all identifiable information removed. The survey received ethical approval from the National Research Ethics Committee of Bangladesh, and the authors were authorized to use the dataset for independent research purposes. Therefore, no additional ethical approval was required.

### Study design

This study employed a cross-sectional design using nationally representative survey data to identify determinants and predict C-section delivery among Bangladeshi women. The analysis focused on socio-demographic and health-related factors influencing delivery mode and machine learning models were applied to predict the likelihood of C-section delivery.

### Dataset source and sample size

The data for this study were obtained from the BDHS 2022, conducted under the authority of the National Institute of Population Research and Training (NIPORT), Ministry of Health and Family Welfare of Bangladesh. The survey was executed by Mitra and Associates, a research firm, with financial support from the Government of Bangladesh and the United States Agency for International Development (USAID). Data collection took place between June 27 and December 12, 2022, and the dataset is publicly accessible online. A two-stage stratified cluster sampling method was employed to ensure a representative sample. In the first stage, 675 enumeration areas (EAs) were selected from a comprehensive list covering the entire country, with 438 EAs from rural regions and 237 EAs from urban regions, selected probability proportional to their size. A complete household listing was then created to serve as a sampling frame for the second stage. In the second stage, a systematic sample of 45 households per EA was selected, resulting in a sample capable of providing reliable socio-demographic and health-related estimates for both rural and urban areas across the eight administrative divisions of Bangladesh. The survey targeted ever-married women aged 15–49 years, who were interviewed face-to-face using a structured questionnaire. Respondents provided detailed information about their reproductive history, health behaviors, and socio-demographic characteristics. A total of 30,375 households were successfully interviewed, resulting in 64,724 individual records of ever-married women aged 15–49 years and these records were used as the initial dataset for the present analysis.

**Target Variables** The target variable for this study is the mode of delivery, specifically focusing on C-section deliveries. Mothers were asked whether their most recent childbirth was delivered through a C-section. For the purpose of analysis, the target variable was coded as follows: 0 for “Vaginal Delivery” and 1 for “C-Section Delivery.”

**Predictor Variables** This study identified several predictor variables based on insights from previous research [24–27]. These predictors include maternal age, age at 1^st^ birth, place of residence, religion, maternal education, partner’s education, employment status, wealth status, parity, number of births in the past year, history of pregnancy termination, pregnancy duration, birth interval, gravidity, occurrence of twin births, child’s sex, ANC visit, maternal Body Mass Index (BMI), baby’s birth weight, and place of delivery. Detailed information about each predictor, including their data types, descriptions, and categorizations, is presented in S1 Table.

### Data preprocessing

Data preprocessing is a critical step performed before to model training to enhance the efficiency and accuracy of machine learning algorithms [28]. Among the 64,724 samples, 5,331 women reported their mode of delivery while the remaining records lacked information on this outcome variable and were therefore excluded. From these 5,331 respondents, only those with complete information for all selected predictor variables were retained and resulted in 2,551 valid cases for analysis. Since the focus of this study was to develop machine learning models based on complete cases, no imputation was performed for missing values. To further improve data quality, outliers in continuous variables were identified and treated using the Interquartile Range (IQR) method which is a robust approach. Observations identified as extreme outliers were excluded rather than transformed or winsorized to avid introducing artificial values that could distort the performance of ML models. This approach ensured that the dataset remained representative of realistic variation in the population while maintaining data quality and consistency. Following preprocessing, the final dataset comprised 2,490 complete observations which were used for model development and subsequent analyses. To enhance transparency and reproducibility, a summary of the data preprocessing steps is provided in S2 Table.

### Feature scaling

Feature scaling is an essential step in the preprocessing of data that standardizes all features to a similar scale, ensuring that no single feature disproportionally influences the ML models performance [28]. Effective feature scaling allows training and tuning processes to operate efficiently, enhancing the models accuracy and stability [29]. Common scaling techniques include min-max scaling, standard scaling, max absolute scaling, and robust scaling [30]. In this study, we applied min-max scaling to transform the features to a range between 0 and 1, as shown in the formula below:


$$X_{scaled}\;=\frac{{X-X}_{min}}{X_{max}-X_{min}}$$

where, *X* is an input feature, *X*_min_ is the minimum value of *X, X*_max_ is the maximum value of *X* and *X*_scaled_ is the scaled output. Min–max scaling was selected because it preserves the original distribution and relative distances of feature values. This approach is particularly beneficial for distance- and margin-based algorithms such as KNN, SVM, and LR. It also remains fully compatible with tree-based methods including DT, RF, and XGB which are inherently scale-invariant. Categorical features encoded numerically were excluded from scaling because their values represented discrete classes rather than continuous magnitudes. To prevent data leakage, scaling was performed after splitting the dataset into training and testing sets. Parameters derived from the training set were not influenced by the test data.

### Feature selection

Feature selection is a crucial step in machine learning that identifies the most relevant and informative attributes for classification, helping to reduce data dimensionality, enhance prediction accuracy, and prevent issues like overfitting, underfitting and excessive computational complexity [29,31]. In this study, the C-section dataset initially included 20 attributes (16 categorical and 6 numerical). Utilizing all features in model training could introduce noise and prolong computation time. To address this, we applied three feature selection techniques: RFE, BFS, and RF. RFE is a wrapper method that recursively eliminates less significant features to retain the most important ones [32]. BFS, an RF-based technique, compares real features with random “shadow” features, providing a robust, unbiased selection process in high-dimensional data [33]. RF constructs multiple decision trees and ranks features based on their contribution to the model’s performance [34]. The initial set of 20 features was selected based on prior literature and domain knowledge in maternal health. This ensured the inclusion of variables known to influence C-section delivery performance. The final selected features were defined as the intersection of features identified by each method:


$$F_{final}\;=F_{RFE}\;\cap \;F_{BFS\;}F\cap \;F_{RF\;importance}$$

here *F*_RFE_, *F*_BFS_ and *F*_RF_ represent the feature sets selected by RFE, BFS and RF, respectively. A total of 10 features were consistently identified across all three methods and were retained for model training, minimizing noise and improving interpretability (see S4 Tabl**e**).

### Machine learning algorithms

In this study, we utilized several widely recognized ML algorithms, including LR, SVM, KNN, DT, RF, and XGB, to predict C-section deliveries based on associated risk factors. This diverse selection was made to balance interpretability, methodological diversity, and prior evidence of effectiveness in health-related prediction tasks. LR served as a baseline linear model suitable for binary classification, offering interpretability through coefficients and odds ratios [35,36]. SVM was used to construct an optimal hyperplane that maximizes the margin between classes, making it effective for high-dimensional data [29]. KNN, a simple instance-based method, classified cases based on the Euclidean distance to the nearest neighbors [36]. DT applied a hierarchical tree-like structure with decision rules at internal nodes and outcomes at leaf nodes to categorize delivery modes [37]. RF, an ensemble of decision trees, aggregated predictions through majority voting to enhance robustness and reduce overfitting [31,38,39]. Finally, Extreme Gradient Boosting (XGB) combined boosting techniques with decision trees by iteratively refining errors of previous models, thereby achieving superior predictive performance [40–42]. These algorithms were chosen to capture both linear and nonlinear relationships. They also allow comparison between simple and ensemble approaches and provide a robust evaluation of predictive accuracy for C-section deliveries.

### Data Partition

This study included 2,490 samples and among them, C-section is 45.6% (*n* = 1135) and vaginal delivery 54.4% (*n* = 1355). The dataset was partitioned into two sets: 70% for training (C-section: 45.6% (*n* = 794), vaginal delivery: 54.4% (*n* = 949) and 30% for testing (C-section: 45.6% (*n* = 341), vaginal delivery: 54.4% (*n* = 406). Stratified random sampling was applied to preserve the proportional representation of each class. To ensure consistency, the class distribution before and after partitioning was visualized using a bar diagram (see S1 Fig). The hold-out test set was entirely excluded from model training and hyperparameter tuning and was solely used to provide an unbiased evaluation of final model performance, including the computation of AUC-ROC curves.

### Cross-validation and hyper-parameter tuning

In this study six machine learning algorithms (LR SVM KNN DT RF and XGB) were employed. Each algorithm required careful adjustment of hyperparameters to optimize performance. To determine the optimal settings, we applied a grid search combined with repeated stratified 10-fold cross-validation. The training dataset was partitioned into 10 folds. Nine folds were used for training and one for validation. This process was repeated 3 times to reduce variability in performance estimates. The best hyperparameters identified during tuning were fixed and applied consistently across all repetitions. This approach helped avoid overfitting and ensured comparability between models. While repeated cross-validation increased computational demands we considered this trade-off necessary to achieve robust and stable performance estimates (Table 1).

**Table 1:** Hyper parameters tuning of different classifiers using GridSearchCV.

Classifier	Hyper-parameter and value
LR	C = **1.0**, penalty = l1, solver = liblinear
SVM	C = 15, gamma = auto, probability = True
KNN	n_neighbors = 15, weights = uniform, metric = manhattan
DT	max_depth = 5, criterion = gini, min_samples_split = 10, min_sample_leaf = 10, max_features = None, random_state = 42
RF	n_estimators = 100, criterion = gini, bootstrap = True, max_depth = 5, max_features = None, min_samples_split = 10, random_state = 42
XGB	n_estimators = 10, booster = gbtree, gamma = 1, min_child_weight = 5

### Performance evaluation

For the binary classification problem, a confusion matrix was employed to evaluate the performance of different ML models. Key performance metrics, including accuracy, precision, recall, F1-score and error rate, were derived from the confusion matrix. These metrics provide a comprehensive assessment of each model’s predictive ability and guide the selection of the best-performing model.

Confusion matrix:

**Table:** 

		*Predicted class*	
		*Positive class(1)*	*Negative class (0)*
*Actual* *class*	*Positive class (1)*	*TP*	*FP*
	*Negative class (0)*	*FN*	*TN*

Performance metrics equations:


$$Accuracy\;=\frac{\left(Tp+\;TN\right)}{\left(TP+TN+FN+FP\right)}$$

$$Precision\;=\frac{TP}{TP+FP}$$

$$\;Recall\;or\;Sensitivity\;=\;\frac{TP}{TP+FN}$$

$$F\text{1}-Score\;=\;\text{2}\frac{Precision\;\times \;Recall}{Precision\;+\;Recall}$$

$$\;Specificity\;=\;\frac{TN}{TN+FP}$$

$$\;Error\;=\;\frac{\left(\;FP+FN\right)}{\left(TP+TN+FN+FP\right)}$$

Where TP, TN, FP, and FN denote true positive, true negative, false positive, and false negative respectively. Two additional metrics such as the area under the curve (AUC), and the receiver operating characteristic (ROC) were also used to evaluate the binary classification models. AUC refers to the area under the ROC curve which plots the trade-off between the true positive rate and the false positive rate across different classification thresholds. The formula for measuring AUC is as follows:


$$AUC=\int_{x=\text{0}}^{\text{1}}\;\text{TPR}\left(FPR^{-\text{1}}(x)\right)dx$$

To rigorously compare the predictive performance of the machine learning models, we applied McNemar’s test to evaluate differences in classification performance between paired models.

### Kernel shape model interpretability

Shapley Additive Explanations (SHAP), introduced by Lundberg and Lee (2017), is an interpretive visualization technique based on Shapley values. This approach explains the contribution of each feature in the ML-based model using Shapley values, which originate from cooperative game theory [43]. Predictors influence the model’s outcome with various magnitudes and directions (positive or negative), as measured by the shapely values.

Shapley values estimate feature importance value and sign of risk factors contributing to the model’s outcome. Shapley values represent the contribution of risk factors to the model’s outcome, assigning feature importance value and its direction (positive or negative sign). In the model, risk factors with positive SHAP values aid in predicting women who undergo C-sections, whereas those with negative SHAP values assist in forecasting women who have normal deliveries. Specifically, the importance of each factor, such as the k^th^ risk factor, is determined by the Shapley values calculated using the following formula.


$$\emptyset_{k}(v)=\frac{\text{1}}{M!}\sum \;S\subseteq M\{k\}\;|S|!(M-|S|-\text{1})![v(S\cup \{k\})-v(S)]$$

Where S represents the subset of risk factors that does not include the risk factor for which the value is being calculated; $\emptyset_{k}(v)$; S∪{k} is the subset of risk factors including k^th^ risk factor in S, v(S) is the outcome of the ML-based model that explains using the risk factors in S; and S⊆M{k} indicates all the sets of S that are subset of the entire set of M risk factors, excluding k^th^ one.

### Software/Tools

**Fig 1:**
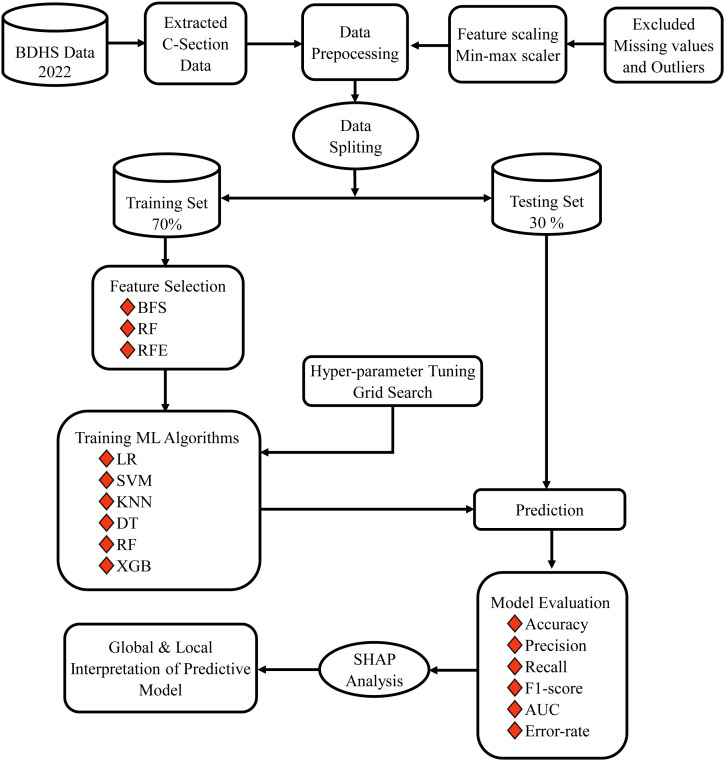
An overview of the comprehensive workflow followed in this study.

All analysis were conducted using Python (version 3.11.10) and R (version 4.3.3). The following libraries were used for data preprocessing, visualization and model development: NumPy and Pandas for data manipulation, Matplotlib ad ggplot2 for plotting, Scikit-learn for implementing ML algorithms and cross-validation, XGBoost for gradient boosting, and SHAP for model interpretability. The workflow of this study is shown in **Fig 1**.

## Results

### Characteristics of the respondents

The final analytic sample included 2,490 complete cases, representing a subset of the original 64,724 records. The study focused on ever-married Bangladeshi women aged 15–49 years, each with at least one child under five years of age (see **Table 2**). The prevalence of C-section at the population level was 45.6%, while 54.4% of women had vaginal deliveries. The average age of the participants was 25.74 ± 5.62 years, and they gave birth to their first child at an average age of 19.31 years. Approximately 68% of the participants resided in rural areas, with 41.1% of these women having undergone C-sections. Nearly one-fifth of the participants had completed higher education, and 70% of them had C-sections. Over 20% of participants’ husbands had a higher education level, and the C-section rate among them was higher (71%) compared to those whose husbands had secondary or lower education or no formal education. The prevalence of C-sections increased with the financial status of the families; 70% of women in the highest wealth quintile underwent C-sections. Women with only one child had a higher C-section rate (52.7%) compared to those with two or more children. The prevalence of C-sections also rose with the number of ANC visits, with 57.9% of women who had three or more ANC visits delivering via C-section. About 72% of women gave birth in a hospital, and of those, 63.4% underwent a C-section.

**Table 2:** Characteristics of the study participants.

Characteristics	Total, n (%) (*n* = 2,490)	Vaginal Delivery, n(%) (*n* = 1355; 54.4%)	C-Section, n(%) (*n* = 1135; 45.6%)
**Maternal age**			
Mean age ± SD	25.74 ± 5.62	25.74 ± 5.70	25.74 ± 5.52
**Age at 1**^**st**^ **birth**			
Age of 1^st^ birth Mean ± SD	19.31 ± 3.61	18.61 ± 3.13	20.13 ± 3.95
**Place of residence**			
Rural	1685 (67.7)	992 (58.9)	693 (41.1)
Urban	805 (32.3)	363 (45.1)	442 (54.9)
**Religion**			
Muslim	2275 (91.4)	1267 (55.7)	1008 (44.3)
Non-Muslim	215 (8.6)	88 (40.9)	127 (59.1)
**Maternal education**			
No education	137 (5.5)	112 (81.7)	25 (18.3)
Primary	550 (22.1)	384 (69.8)	166 (30.2)
Secondary	1314 (52.8)	717 (54.6)	597 (45.4)
Higher	489 (19.6)	142 (29.1)	347 (70.9)
**Partner’s education**			
No education	379 (15.2)	286 (75.5)	93 (24.5)
Primary	732 (29.4)	494 (67.5)	238 (32.5)
Secondary	868 (34.9)	427 (49.2)	441 (50.8)
Higher	511 (20.5)	148 (29.0)	363 (71.0)
**Employment Status**			
Yes	536 (21.5)	316 (59.0)	220 (41.0)
No	1954 (78.5)	1039 (53.6)	915 (46.4)
**Wealth status**			
Poorest	506 (20.3)	396 (78.3)	110 (21.7)
Poorer	500 (20.1)	319 (63.8)	181 (36.2)
Middle	503 (20.2)	269 (53.5)	234 (46.5)
Richer	504 (20.2)	228 (45.2)	276 (54.8)
Richest	477 (19.2)	143 (30.0)	334 (70.0)
**Parity**			
1 birth	928 (37.3)	439 (47.3)	489 (52.7)
2 births	1480 (59.4)	845 (57.1)	635 (42.9)
3 and above	82 (3.3)	71 (86.6)	11 (13.4)
**Number of birth in the past year**			
Yes	1023 (41.1)	544 (53.2)	479 (46.8)
No	1467 (58.9)	811 (55.3)	656 (47.7)
**History of pregnancy termination**			
Yes	512 (20.6)	271 (52.9)	241 (47.1)
No	1978 (79.4)	1084 (54.8)	894 (45.2)
**Pregnancy duration**			
Mean ± SD	8.97 ± 0.40	9.01 ± 0.38	8.93 ± 0.41
**Birth interval**			
Mean ± SD	27.76 ± 15.26	25.80 ± 13.43	30.10 ± 16.85
**Gravidity**			
Premigravida	798 (32.0)	379 (47.5)	419 (52.5)
Multigravida	1529 (61.4)	849 (55.5)	680 (44.5)
Grand multigravida	163 (6.5)	127 (77.9)	36 (22.1)
**Occurrence of twin births**			
Yes	26 (1.0)	13 (50.0)	13 (50.0)
No	2464 (99.0)	1342 (54.5)	1122 (45.5)
**Child Sex**			
Male	1304 (52.4)	711 (54.5)	593 (45.5)
Female	1186 (47.6)	644 (54.3)	542 (45.7)
**ANC visits**			
No visit	191 (7.7)	173 (90.6)	18 (9.4)
1–2 Visits	819 (32.9)	559 (68.3)	260 (31.7)
3 and above visits	1480 (59.4)	623 (42.1)	857 (57.9)
**Maternal body mass index**			
BMI Mean ± SD	22.78 ± 4.15	22 ± 3.84	23.70 ± 4.30
**Baby weight**			
Baby Weight Mean ± SD	2754.26 ± 616	2573.8 ± 536.3	2969.8 ± 635.7
**Place of delivery**			
Home	700 (28.1)	700 (100.0)	0 (0.0)
Hospital	1790 (71.9)	655 (36.6)	1135(63.4)

### Risk factors identification

Feature selection techniques are essential for identifying and selecting the most relevant and informative features from a dataset to enhance model performance. In this study, three feature selection algorithms including RFE, BFS and RF were applied to identify key features associated with C-section delivery. The RFE method identified 14 important features, while BFS selected 13 and RF recognized 11 features (see S3 Table). Ten features were consistently identified across all three methods, indicating their significance as risk factors for C-section delivery (see **Table 3**). These common features, informed by both algorithmic selection and prior maternal health knowledge, were subsequently used in the machine learning models to improve predictive accuracy and interpretability. Features not consistently selected across all three methods were excluded to enhance model robustness and reduce noise from less informative predictors

**Table 3:** Ten key risk factors for C-section delivery were identified by three feature selection techniques.

Variables	Data type	Descriptions	Categorizations
Maternal age	Continuous	Mother’s age in years	--
Age at 1^st^ birth	Continuous	Age of 1^st^ birth of mother	--
Maternal education	Ordinal	Mother’s educational level	i) No education ii) Primary iii) Secondary iv) Higher
Partner’s education	Ordinal	Partner’s Education level	i) No education ii) Primary iii) Secondary iv) Higher
Wealth status	Ordinal	Mother’s wealth index	i) Poorest ii) Poorer iii) Middle iv) Rich v) Richest
Birth interval	Discrete	Marriage to 1^st^ birth interval in months	--
ANC visits	Ordinal	Number of antenatal care visits during pregnancy	i) No visit ii) 1–2 visits iii) 3 and above visits
BMI	Continuous	Maternal body mass index	--
Baby weight	Continuous	Weight of baby in grams	--
Place of delivery	Binary	Place of delivery of baby	i) Home ii) Hospital

### Performance comparison

The performance of the six ML models in predicting C-section delivery using the BDHS dataset is summarized in **Table 4**. The models were evaluated using multiple metrics, including accuracy, precision, recall, F1-score, AUC, and error rate. These metrics provide a comprehensive assessment of each model’s predictive capability and reliability.

**Table 4:** Performance metrics of machine learning models for predicting C-section delivery.

Models	Accuracy	Precision	Recall	F1-Score	Error
LR	78.71	71.16	89.74	79.38	21.29
SVM	77.38	68.94	91.79	78.74	22.62
KNN	77.64	70.81	86.80	77.99	22.36
DT	80.19	70.84	96.19	81.59	19.81
RF	81.79	72.83	95.89	82.78	18.21
XGB	79.92	72.47	90.32	80.42	20.08

**Fig 2:**
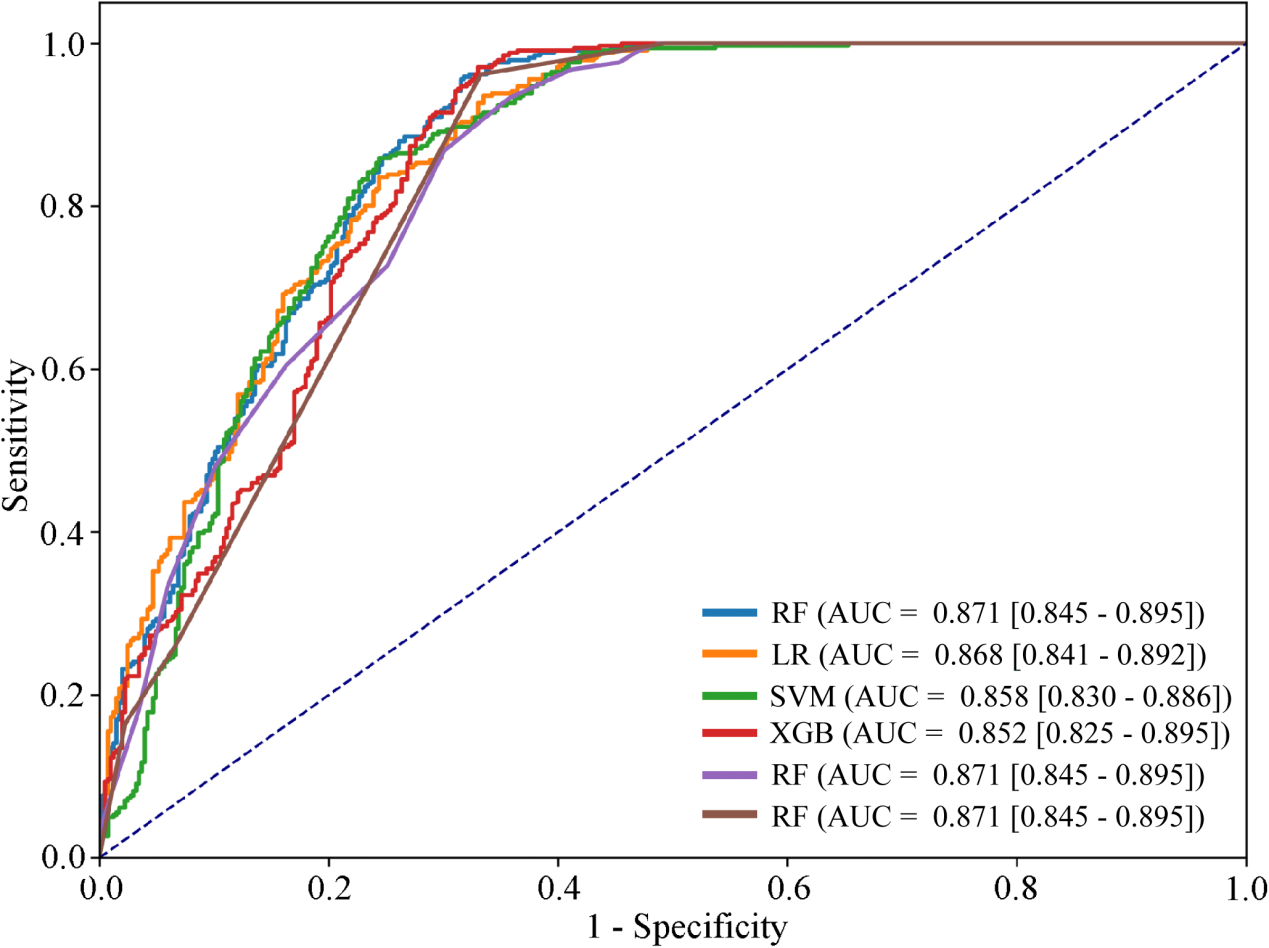
ROC curves of machine leaning models for predicting C-section delivery.

Among the models, the Random Forest (RF) classifier demonstrated the best overall performance, achieving the highest accuracy (81.79%), precision (72.83%), recall (95.89%), and F1-score (82.78%). Its AUROC score of 0.871 (95% CI: 0.845–0.895) further indicates a strong discriminatory ability between C-section and vaginal delivery (see **Fig 2**). The RF model also recorded the lowest error rate (18.21%), underscoring its robustness and predictive capacity. Notably, the high recall highlights its effectiveness in correctly identifying C-section cases, emphasizing its potential clinical utility in maternal health decision-making. Other models, including LR, SVM, KNN, DT, and XGB, showed competitive performance, but their metrics and AUROC values were slightly lower. To rigorously compare model performance, McNemar’s test was applied to all model pairs (S5 Table). RF demonstrated statistically significant superiority over LR (*p* < 0.001), SVM (*p* = 0.0099), and KNN (*p* = 0.036). Additionally, LR significantly outperformed SVC, XGB, KNN, and DT (all *p* < 0.001). These results indicate that RF consistently provided the most robust predictions among the evaluated algorithms.

### Interpretable risk factors of C-section

**Fig 3:**
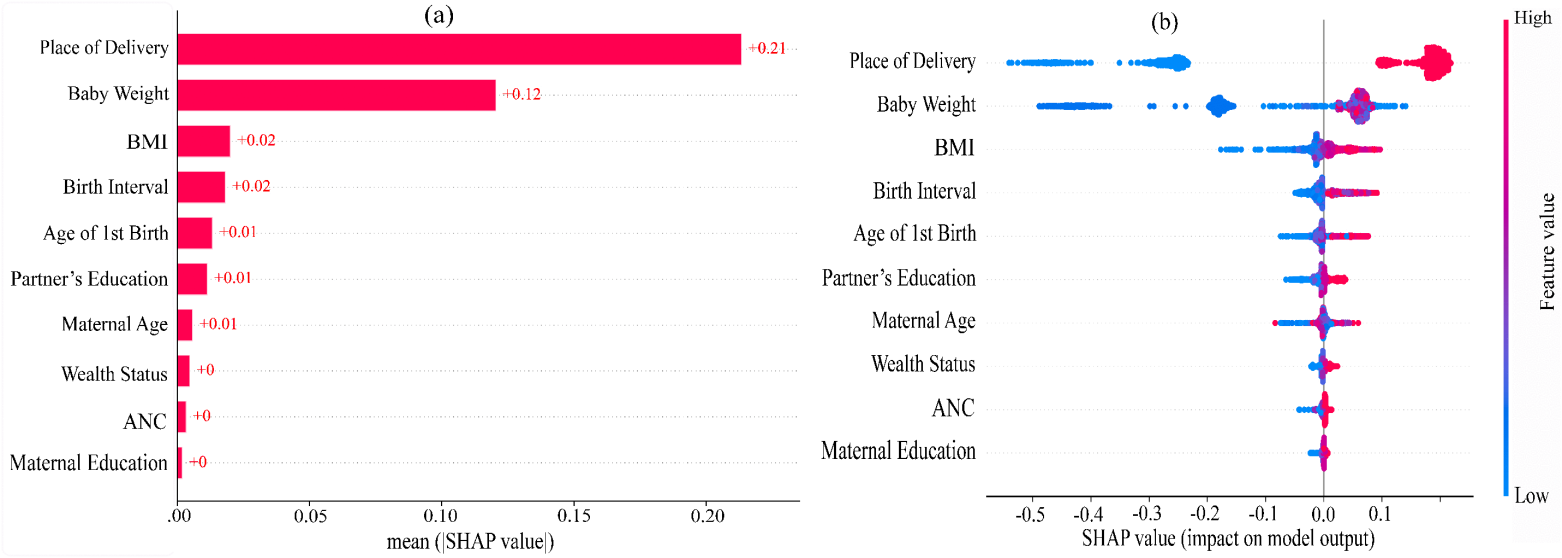
Feature importance of risk factors based on SHAP values, (a) Mean absolute SHAP values (b) Local explanation summary.

An in-depth SHAP analysis was performed within the RF model to identify interpretable socio-demographic risk factors for C-section delivery based on SHAP values. **Fig 3** explains the global importance of each socio-demographic risk factor to the prediction of RF-based model. The global importance plot identifies the impact of the most significant risk factors on the model’s prediction but does not indicate whether they affect the prediction positively or negatively. Therefore, summary plot is employed, which provides a global macro-level explanation of how risk factors contribute to the model’s predictions. The x-axis displays the SHAP values, which represent the direction and magnitude of each risk factor’s influence on the model’s prediction, with positive values indicating an increased risk of C-section and negative values suggesting a decreased risk. The color gradient represents the SHAP value of each risk factor, with blue denoting low values and red indicating high values. The SHAP analysis revealed several significant socio-demographic risk factors associated with C-section delivery such as ‘Place of delivery’, ‘Baby Weight’, maternal BMI’, ‘Birth interval’, ‘Age of 1^st^ birth’, ‘Partner’s education’, ‘Maternal age’, ‘Wealth status’, ‘ANC visits’ and ‘Maternal education’. ‘Place of delivery’, ‘Birth weight’, ‘maternal BMI’, and ‘Birth interval’ are strongly correlated with increased C-section delivery while remaining risk factors also elevate this risk.

## Discussion

This study aimed to identify socio-demographic risk factors and predict C-section delivery among ever-married women aged between 15 and 49 years in Bangladesh. The analysis revealed that 45.6% of the participants delivered via C-section, a rate that significantly exceeds the WHO’s recommended threshold of 10–15% for any population [3]. This sharp increase is consistent with the rising trend observed in Bangladesh where C-section rates increased from 24% in 2014 to 32% in 2017 [24,27]. The current rate reported in our study is the highest ever recorded in Bangladesh, reflecting the growing reliance on C-sections, possibly influences by sociocultural and healthcare-related factors.

Using BDHS data, this study included 2,490 participants and analyzed 20 features potentially associated with C-section delivery. By applying BFS, RFE, and RF feature importance, we identified 10 significant predictors and used them to train six ML models: LR, SVM, KNN, DT, RF and XGB. Among these, the RF classifier demonstrated the best overall performance (accuracy 81.79%, AUC 0.871), with a notably high recall of 95.89%, emphasizing its effectiveness in correctly identifying C-section cases. McNemar’s test confirmed that RF significantly outperformed LR, SVM, and KNN, while performing comparably to XGB and DT. In healthcare contexts, where minimizing false negatives is crucial, RF’s high recall combined with statistically significant superiority highlights its practical utility for C-section prediction. The slightly lower accuracy of our RF model compared to previous studies (e.g., 83.7% in BDHS 2017–18 using Naïve Bayes [23] and 84.0% accuracy observed in a Korean cohort study using the XGB model [44]. However, our RF model outperformed studies with reported accuracies below 71.0% for C-section prediction [45]. Therefore, the main novelty of our work lies not in marginal improvements in predictive power but in the integration of SHAP interpretability to provide transparent, clinically meaningful insights into model predictions.

The SHAP analysis of the RF model identified place of delivery, baby weight, BMI, birth interval, age at first birth, partner’s education and maternal age as significant predictors for C-section delivery. The place of delivery emerged as the most influential factor, consistent with findings from previous studies using BDHS data, where institutional deliveries were strongly associated with higher C-section rates [23]. In South and South-East Asian regions, place of delivery is found to be a crucial factor that influences the decision to have a C-section delivery [2]. Baby weight was identified as another major contributor to C-section delivery. High baby weight often leads to complications during vaginal delivery, necessitating a surgical approach. A study conducted in China similarly observed increased C-section rates among infants with higher birth weights [46]. The influence of BMI and maternal age at first birth as predictors aligns with previous research that identified these factors as significant in predicting delivery mode [24]. Notably, maternal age has been linked to increased obstetric complications, including hypertension, gestational diabetes, and malposition, which may necessitate C-sections [47]. Previous research also found that advanced maternal age is a strong determinant of C-sections due to associated health risks and healthcare professionals’ preference for surgical delivery to avoid complications [48,49].

Socioeconomic status emerged as a significant determinant. Women from wealthier households had higher C-section rates, likely due to their financial ability to afford surgical interventions and a preference for avoiding labor pain [15,50,51]. Additionally, the number of ANC visits was strongly associated with C-section rates. Women who attended three or more ANC visits had a higher likelihood of undergoing a C-section. While ANC visits are generally aimed at promoting maternal health, evidence suggests that healthcare providers may often recommend C-sections during these visits, particularly in private healthcare settings [52,53]. This finding supports previous literature that suggests frequent ANC visits are linked to increased C-section rates [54]. Interestingly, both maternal and paternal education levels were significantly associated with C-section deliveries. Higher education often translates to better health literacy, access to private healthcare facilities, and the capacity to make informed choices regarding delivery methods [24,50]. The findings provide actionable insights for maternal health policy in Bangladesh. By identifying key predictors, policymakers can target high-risk groups with tailored interventions such as enhanced prenatal counseling, stricter monitoring of C-section rates in private hospitals and promotion of evidence-based delivery practices. These measures align with national health priorities, including improving safe delivery practices and reducing maternal and neonatal morbidity. Predictive insights from ML models can further guide resource allocation, optimize prenatal screening and inform preventive strategies, ensuring interventions are efficient and equitable. The internal validity of our findings is strengthened by the large, nationally representative dataset, rigorous feature selection, and evaluation using multiple complementary performance metrics alongside SHAP interpretability [55,56]. External validity is more limited, as the model was developed using data specific to Bangladeshi women. Generalizability may be restricted due to differences in healthcare systems, sociocultural practices, and maternal health characteristics. Future research should validate these models in diverse settings, including other LMICs, and employ prospective or clinical datasets to assess real-world predictive performance [57,58].

## Limitations of the Study

Despite the valuable insights provided, several limitations should be acknowledged. The cross-sectional design restricts causal inference. While complete case analysis was applied, future studies could explore alternative missing data strategiess such as multiple imputations to assess their impact on model performance. Reliance on self-reported data may introduce recall bias. Importantly, several key clinical predictors were unavailable in BDHS 2022, including maternal comorbidities (e.g., hypertension, gestational diabetes), detailed obstetric history and provider-related information (e.g., hospital type, decision-making authority) which may limit predictive comprehensiveness. Additionally, although hyperparameter tuning and repeated cross-validation were performed, the RF model could still be prone to overfitting. Future research should consider regularization or pruning techniques to enhance model robustness and generalizability.

## Conclusions

This study applied machine learning techniques to identify determinants and predict C-section delivery among ever-married Bangladeshi women aged 15–49 years using the nationally representative BDHS 2022 data. Ten key socio-demographic factors including place of delivery, baby weight, BMI, birth interval, maternal age, age at first birth, partner’s education, ANC visits, socioeconomic status and maternal education were identified as significant predictors of C-section delivery. Among the evaluated models, the RF classifier demonstrated the highest predictive performance highlighting its potential utility for accurately identifying high-risk cases. These findings underscore the growing reliance on C-sections in Bangladesh and the importance of data-driven approaches to inform clinical decision-making. From a policy perspective, targeted interventions such as monitoring C-section rates in private healthcare facilities, enhancing maternal counseling and promoting evidence-based guidelines could help reduce unnecessary procedures and improve maternal and neonatal health outcomes. Future research should consider integrating clinical data, employing prospective cohort designs and validating models in diverse populations to strengthen causal inferences and generalizability. Overall, this study demonstrates the potential of ML to support maternal health policy and improve healthcare planning in resource-limited settings.

## Supporting information

S1 Table Description of socio-demographic variables. (DOCX)

S2 Table Summary of data processing steps.\ (DOCX)

S3 Table Feature identified by three feature selection techniques. (DOCX)

S4 Table Top features ranking selected by three feature selection techniques. (DOCX)

S5 Table McNemar test: Used to make a performance comparison between two algorithms. (DOCX)

S1 Fig Class distribution of delivery modes full, before and after dataset partitioning. (TIF)

S1 Data Code. (TXT)
