# Supplementary material for: Identifying determinants and predicting cesarean section delivery among Bangladeshi women using machine learning: Insight from BDHS 2022 Data
Source: PLOS Glob Public Health. 2025 Nov 19;5(11):e0005494. doi: 10.1371/journal.pgph.0005494 (PMC12629447; doi:10.1371/journal.pgph.0005494)
Supplement: S1 Table — (DOCX) [file pgph.0005494.s001.docx]

**S1 Table:** Description of socio-demographic variables

| **SN** | **Variables** | **Data type** | **Descriptions** | **Categorizations** |
| --- | --- | --- | --- | --- |
| 1 | Mother’s Age | Continuous | Mother’s age in years | -- |
| 2 | Age of 1^st^ Birth | Continuous | Age of 1^st^ birth of mother | -- |
| 3 | Residence | Nominal | Type of place of residence | 1. Rural 2. Urban |
| 4 | Religion | Nominal | Religion | 1. Islam 2. Others |
| 5 | Education | Ordinal | Mother’s educational level | 1. No education 2. Primary 3. Secondary 4. Higher |
| 6 | Partner’s Education | Ordinal | Partner’s Education level | 1. No education 2. Primary 3. Secondary 4. Higher |
| 7 | Working Status | Binary | Working status | 1. Yes 2. No |
| 8 | Wealth | Ordinal | Mother’s wealth index | 1. Poorest 2. Poorer 3. Middle 4. Rich 5. Richest |
| 9 | Parity | Ordinal | Parity at sterilization | 1. 1 birth 2. 2 births 3. 3 and above births |
| 10 | Birth in past year | Binary | Mother gave birth last year | 1. Yes 2. No |
| 11 | Terminated | Binary | Ever terminated pregnancy | 1. Yes 2. No |
| 12 | Pregnancy Duration | Discrete | Duration of pregnancy in months | -- |
| 13 | Birth Duration | Discrete | Marriage to 1^st^ birth interval in months | -- |
| 14 | Gravidity | Ordinal | Total number of pregnancies | 1. Primigravida 2. Multigravida 3. Grand multigravida |
| 15 | Twin | Binary | Baby is twin | 1. Yes 2. No |
| 16 | Child-Sex | Nominal | Sex of child | 1. Male 2. Female |
| 17 | ANC | Ordinal | Number of antenatal care visits during pregnancy | 1. No visit 2. 1-2 visits 3. 3 and above visits |
| 18 | BMI | Continuous | Body mass index | -- |
| 19 | Baby Weight | Continuous | Weight of baby in grams | -- |
| 20 | Delivery Place | Binary | Place of delivery of baby | 1. Home 2. Hospital |
