# Supplementary material for: Identifying determinants and predicting cesarean section delivery among Bangladeshi women using machine learning: Insight from BDHS 2022 Data
Source: PLOS Glob Public Health. 2025 Nov 19;5(11):e0005494. doi: 10.1371/journal.pgph.0005494 (PMC12629447; doi:10.1371/journal.pgph.0005494)
Supplement: S2 Table — (DOCX) [file pgph.0005494.s002.docx]

**S2 Table:** Summary of data processing steps.

| **Steps** | **Number of Records** | **Records Removed** | **Remaining Records** |
| --- | --- | --- | --- |
| Original dataset | 64,724 | - | 64,724 |
| Valid responses for target variable | - | 59,393 | 5,331 |
| Complete case analysis (all predictors) | - | 2,780 | 2,551 |
| Outlier removal (continuous variables) | - | 61 | 2,490 |
| Final dataset for ML | - | - | 2,490 |
