# Supplementary material for: Identifying determinants and predicting cesarean section delivery among Bangladeshi women using machine learning: Insight from BDHS 2022 Data
Source: PLOS Glob Public Health. 2025 Nov 19;5(11):e0005494. doi: 10.1371/journal.pgph.0005494 (PMC12629447; doi:10.1371/journal.pgph.0005494)
Supplement: S3 Table — (DOCX) [file pgph.0005494.s003.docx]

**S3 Table:** Feature identified by three feature selection techniques

| **Feature selection technique** | **Selected Features** |
| --- | --- |
| Recursive Feature Elimination (RFE) | Mother's Age, Age of 1st birth, Residence, Education, Wealth, Parity, Birth in past year, Gravidity, BMI, Partner's Education, ANC, Baby Weight, Birth Duration, and Delivery place |
| Brouta | Delivery place, Baby Weight, Partner's Education, BMI, Age of 1st birth, Birth Duration, Wealth, ANC, Mother's Age, Education, Gravidity, Parity, Pregnancy Duration' |
| Random Forest (RF) | Delivery place, Baby Weight, BMI, Birth Duration, Age of 1st birth, Partner's Education, Wealth, Education, ANC, Mother's Age and Residence. |
