# Supplementary material for: Identifying determinants and predicting cesarean section delivery among Bangladeshi women using machine learning: Insight from BDHS 2022 Data
Source: PLOS Glob Public Health. 2025 Nov 19;5(11):e0005494. doi: 10.1371/journal.pgph.0005494 (PMC12629447; doi:10.1371/journal.pgph.0005494)
Supplement: S4 Table — (DOCX) [file pgph.0005494.s004.docx]

**Table S4:** Top features ranking selected by three feature selection techniques

| Selected feature | RF | RFE | BFS |
| --- | --- | --- | --- |
| Mother’s Age | 0.034 | 1 | 6 |
| Age of 1^st^ Birth | 0.046 | 1 | 2 |
| Education | 0.037 | 1 | 7 |
| Partner’s Education | 0.044 | 1 | 1 |
| Wealth | 0.043 | 1 | 4 |
| Birth Duration | 0.051 | 1 | 3 |
| ANC | .035 | 1 | 5 |
| BMI | .069 | 1 | 1 |
| Baby Weight | 0.246 | 1 | 1 |
| Delivery Place | 0.325 | 1 | 1 |
| Parity | 0.010 | 1 | 9 |
| Residence | 0.031 | 1 | 10 |
| Gravidity | 0.0091 | 1 | 8 |
| Child-sex | 0.0088 | 2 | 12 |
| Birth in past year | 0.0079 | 1 | 13 |
| Pregnancy Duration | 0.0061 | 3 | 10 |
| Work | 0.0060 | 7 | 14 |
| Terminated | 0.0050 | 5 | 15 |
| Religion | 0.004 | 6 | 15 |
| Twin | .0013 | 4 | 17 |

[Note: For random forest feature selection threshold 0.03, For RFE, where ranking value equal to 1 those being selected, For Brouta top 13 were selected for lower to higher values]
