# Supplementary material for: Identifying determinants and predicting cesarean section delivery among Bangladeshi women using machine learning: Insight from BDHS 2022 Data
Source: PLOS Glob Public Health. 2025 Nov 19;5(11):e0005494. doi: 10.1371/journal.pgph.0005494 (PMC12629447; doi:10.1371/journal.pgph.0005494)
Supplement: S5 Table — (DOCX) [file pgph.0005494.s005.docx]

**Table S5:** McNemar test: Used to make a performance comparison between two algorithms

Hypothesis:

*H*_0_: Model 1 performance is different from Model 2
*H*_1_: Model 1 is not different from Model 2, or they performance are the same.

| Comparisons | Test statistic | P-value | Comment |
| --- | --- | --- | --- |
| RF vs LR | 85.1075 | 0.0000 | Statistically significant difference in performance |
| RF vs. SVC | 6.6489 | 0.0099 | Statistically significant difference in performance |
| RF vs. XGB | 0.9730 | 0.3239 | No statistically significant difference in performance |
| RF vs. KNN | 4.4083 | 0.0358 | Statistically significant difference in performance |
| RF vs. DT | 2.2857 | 0.1306 | No statistically significant difference in performance |
| LR vs. SVC | 64.4075 | 0.0000 | Statistically significant difference in performance |
| LR vs. XGB | 84.9412 | 0.0000 | Statistically significant difference in performance |
| LR vs. KNN | 71.6005 | 0.0000 | Statistically significant difference in performance |
| LR vs. DT | 79.6199 | 0.0000 | Statistically significant difference in performance |
| SVC vs. XGB | 3.2079 | 0.0733 | No statistically significant difference in performance |
| SVC vs. KNN | 0.0132 | 0.9087 | No statistically significant difference in performance |
| SVC vs. DT | 4.3011 | 0.0381 | Statistically significant difference in performance |
| XGB vs. KNN | 2.1157 | 0.1458 | No statistically significant difference in performance |
| XGB vs. DT | 0.0250 | 0.8744 | No statistically significant difference in performance |
| KNN vs. DT | 2.7227 | 0.0989 | No statistically significant difference in performance |
